# Properties of Cement Mortar by Use of Hot-Melt Polyamides as Substitute for Fine Aggregate

**DOI:** 10.3390/ma8063714

**Published:** 2015-06-19

**Authors:** Xiongzhou Yuan, Weiting Xu, Wei Sun, Feng Xing, Weilun Wang

**Affiliations:** 1 Guangdong Provincial Key Laboratory of Durability for Marine Civil Engineering, College of Civil Engineering, Shenzhen University, Shenzhen 518060, Guangdong, China; E-Mails: yuanxiongzhou@gmail.com (X.Y.); xingf@szu.edu.cn (F.X.); wwl0610@szu.edu.cn (W.W.); 2 Shenzhen Institute of Information Technology, Shenzhen 518172, Guangdong, China; 3 School of Materials Science and Engineering, Southeast University, Nanjing 211189, Jiangsu, China; E-Mail: sunwei@seu.edu.cn

**Keywords:** hot-melt polyamide, heat treatment, self-repairing, hot-melt polyamide (HMP)/cement composite system, physical adhesion

## Abstract

This paper presents an experimental study on use of hot-melt polyamide (HMP) to prepare mortar specimens with improved crack healing and engineering properties. The role of HMP in the crack repairing of cement mortar subjected to several rounds of heat treatment was investigated. Compatibility between HMP and hydraulic cement was investigated through X-ray diffraction (XRD) and Fourier transform infrared spectra (FTIR) technology. Mortar specimens were prepared using standard cement mortar mixes with HMP at 1%, 3% and 5% (by volume) for fine aggregate substitute. After curing for 28 days, HMP specimens were subjected to heating at temperature of 160 °C for one, two, and three days and then natural cooling down to ambient temperature. Mechanical and durability properties of the heated HMP mortars were evaluated and compared with those of the corresponding mortars without heating. The microscopic observation of the interfacial transition zone (ITZ) of HMP mortar was conducted through environmental scanning electron microscopy (ESEM). Results reveal that incorporation of HMP improves the workability of the HMP/cement binder while leading to decrease in compressive strength and durability. The heated HMP mortars after exposure to heating for one, two, and three days exhibit no obvious change in compressive strength while presenting notable increase in flexural strength and durability compared with the corresponding mortars without heating. The XRD, FTIR and ESEM analyses indicate that no obvious chemical reaction occurs between HMP and hydraulic cement, and thus the self-repairing for interfacial micro-crack in HMP/cement composite system is ascribed to the physical adhesion of HMP to cement matrix rather than the chemical bonding between them.

## 1. Introduction

Cementitious materials are the most widely used building materials all over the world, attributed to its excellent mechanical properties and relatively low cost. However, they are prone to crack formation, even from the very beginning of its service life. These cracks endanger the durability of cementitious materials and concrete structures as aggressive liquids and gasses may enter via these cracks and lead to degradation. The repair works increase the cost of concrete structures as they are labor intensive, and the structures are in disuse during repair [1,2]. Thus, self-healing of the affected cementitious materials is of great importance.

Self-healing phenomenon in cementitious materials has been noticed and studied for a long time. In order to endow or enhance the natural self-healing property of cementitious materials [3,4,5], many different and innovative strategies have been proposed and developed during the past decades. Different strategies employed for the self-healing of cementitious materials have been explored and are summarized as follows: the use of hollow fibers, encapsulation, expansive agents and mineral admixtures, bacteria and shape memory materials. (1) For the use of hollow fibers, the major advantage is that additional supply of healing agent is possible, thus a large amount of healing agent can be handled, which also ensures the effectiveness of this system under multiple damage events. Additionally, the mechanical properties can be expected to be recovered at a high ratio after healing from damage [6,7,8,9,10]. While the major disadvantage is that the casting of concrete becomes difficult. Thus, it may become a severe problem if large amount of concrete needs to be cast on the construction site [11,12,13]. (2) As for the use of capsules, the treatment is easy and they can respond to fracture at many different locations, because they are dispersed inside the matrix [14,15,16,17]. However, a successful manufacture of desirable capsules for the application in cementitious materials is often not that straightforward. Moreover, the healing agent that can be handled in this carrying system is smaller than the system mentioned earlier. Besides, the bond between the capsules and the matrix is often a concern. If the strength of the capsule wall is higher than the bond strength, the capsules would not rupture after the initiation of the cracks, then no healing agent will be released and no healing of the cracks will happen [18,19,20,21]. (3) For the expansive agent and mineral admixtures, due to the expansive characteristic after introduction into cementitious materials, expansive agents, sometimes combined with other mineral additions and/or admixtures, which could definitely improve the self-healing capacity, if appropriately used [22,23]. If an expansive agent without any protection is pre-mixed with cement, then after the addition of water, the expansive agent will hydrate simultaneously with the cement, sometimes even quicker than cement particles to form expansive hydrates depending on the components. However, unexpected expansion may often happen more than in cementitious matrices if the restraint is not appropriately set, which could lead to the immature failure from the inside of the matrices resulting in lowering of strength or even no strength at all [24,25]. (4) Adopting bacteria induced carbonate precipitation to fill the cracks is very innovative and this method, which is a result of biological activities, is pollution free and natural [26,27]. The microbial precipitation depends on several factors, including: the concentration of dissolved inorganic carbon, the pH, the bacterial strain, the concentration of calcium ions and the presence of nucleation sites. Also, when bacteria are used to work for the healing of cracks in concrete, the major hindering factor is the high alkaline environment of concrete, restricting the growth of the bacteria [28,29,30,31]. (5) For the shape memory materials, the relatively high cost makes their use unviable for all but the most specialized of applications, since concrete material is often used in huge amount in practice and hence very cost-sensitive [32,33]. Thus, it would lead to uncertainties of adopting this method.

An ideal self-healing system is supposed to be readily available and cost-effective. A suitable agent should be capable of being readily released and sufficiently mobile to allow migration to the areas where damage occurs. Additionally, crack reopening should be resisted post-healing, and therefore the healed parts should have sufficient mechanical properties after curing, ideally equal to or greater than that of the cementitious matrix. In order to improve long-term durability, the agent should also have sufficient longevity and compatibility with the cementitious matrix over the lifetime of the structure.

Hot-melt adhesives have become very popular in the last decades because they are convenient and satisfy the environment with high viscosity for structure bonding. Hot-melt adhesives are 100% solid thermoplastic compounds that contain neither solvent nor an aqueous carrier for the active adhesive components. These adhesives are solids at room temperature, but they liquefy when heated to the temperature at which they are applied [34]. When applied, hot-melts bond and cool rapidly. With the development of the petrochemical industry, various polymer materials and thermoplastics, such as hot-melt polyamide (HMP), ethylene vinyl acetate copolymers, polyolefins, polyurethane and polyesters, have formed the basis of hot-melt adhesives.

Hot melt polyamides (HMP) has a relatively low melt point and good adhesion to a variety of materials, and its price is low. Thus, it is one of the most popular polymer used in hot-melt adhesives. Generally, HMP for hot-melt adhesive is with softening point in the range of 106°C to 150 °C and has excellent bonding with inorganic material. The HMP is liquefied in melt temperature range and capable of penetrating along the cracks or capillary pores in the solid material and hence coheres to the cracks or pores after cooling down to room temperature.

Our research group proposed a self-healing composite system, a concrete beam containing HMP with optical fibers and shape memory alloys (SMA) embedded in the concrete. Once cracks occur and are detected by optical fibers, SMA will be heated by means of electrifying. When temperature goes up to the martensite start temperature (MS), SMA start to impose compressive force on concrete to force cracks to close. Besides, when the temperature reaches the melting point, HMP will soften and melt into liquid, which is allowed to diffuse into crack through its gravity, capillary action and restrained recovery force.

This research is a primary study of the self-healing composite system, aiming to explore the feasibility of using HMP as a healing agent for concrete crack repairing or modifying after repeated heat treatment. Mortar specimens were prepared with HMP at various mixing dosage (by volume) as substitute for fine aggregate. Strength and durability properties of HMP blended mortars with exposure to various heating durations were evaluated. Compatibility between HMP and hydraulic cement was also investigated through XRD, FTIR and ESEM technology. The crack self-healing performance and the suggestive dosage of HMP were determined based on the consideration of compatibility, mechanical, post-healing, and durability performances of the HMP-cement composite system.

## 2. Research Plan

The experimental program in this study involved four main tasks:

(1) Investigation of melt viscosity, chemical stability and heating-melted adhesion performance of HMP to cement mortars with exposure to 10-minute, 1-day, 2-day and 3-day heating.
(2) Investigation of the compatibility between HMP and hydraulic cement through determining mineralogy by XRD and chemical bonding properties by FTIR of HMP blended paste.
(3) Investigation of fluidity, strength and durability properties of HMP blended mortar.
(4) Investigation of recovery of strength and durability properties of HMP blended mortar after exposure to 1-day, 2-day and 3-day heat treatment, and the microscopic observation (ESEM) of HMP blended mortars after heating for two days.

## 3. Experimental Program

### 3.1. Materials

**Figure 1:**
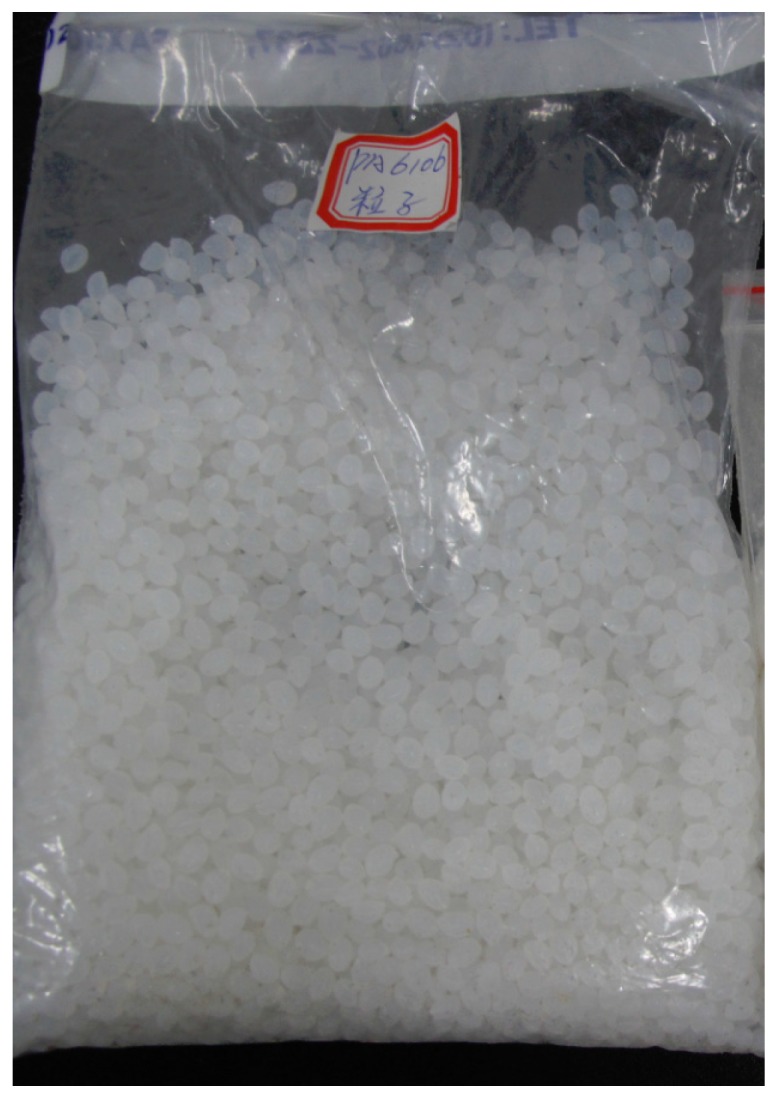
Image of hot-melt polyamide (HMP).

A JCC-61069 HMP with melting point of 106 °C to 116 °C supplied by the Union Camp company was used in this study. It was in the form of spheroid with size of Φ3 × 3 mm, as shown in Figure 1. The physical properties are listed in Table 1.

**Table 1:** Characteristics of hot-melt polyamide (HMP).

Color		Ivory
Melting temperature range (°C)		106–116
Melt Index (±3 g/10 min, 2.16 kg, 160 °C)		60 ± 5
Melt Viscosity (Pa·s/150 °C)		82
Peel strength (N/cm)		≥10
Conglutinate Condition	Temperature (°C)	120–160
	Curing force (kg/cm^2^)	0.1–1.5
	Curing time (s)	10–15

An ordinary Portland cement (CEM I 52.5R) was used in this study. Its initial and final setting time was according to BS EN 196-3:2005+A1:2008 [35] and tested to be 132 and 187 min, respectively, after water addition, and the 28-day compressive strength was tested to be 64.3 MPa according to BS EN 196-1:2005 [36]. The chemical compositions and physical properties of cement are experimentally tested and presented in Table 2.

**Table 2:** The chemical compositions and physical properties of cement.

Chemical composition (% w/w)									
SiO_2_	Al_2_O_3_	Fe_2_O_3_	CaO	MgO	SO_3_	NaO	K_2_O	L.O.I.
20.4	4.8	2.9	63.9	1.5	2.0	0.2	0.4	2.95
Physical characteristics								
Specific surface area (m^2^/kg)						369.6		
Density (g/cm^3^)						3.1		
Initial setting time (min)						132		
Final setting time (min)						187		
28-day compressive strength (MPa)						64.3		
28-day flexural strength (MPa)						9.3		

Fine aggregate used in this study; it was graded river sand passed through a 1.18 mm sieve. Its fineness modulus and specific gravity were measured to be 2.84 and 2.52, respectively.

### 3.2. Melt Viscosity, Chemical Stability and Heating-Melt Adhesive Strength of HMP

Prior to mixing mortars, melt viscosity, chemical stability and heating-melt adhesive strength of HMP to cement mortars after heat treatment were evaluated by melt viscosity test, FTIR and bonding strength of cement mortars.

The melt viscosity of HMP was determined referring to the Chinese standard GB/T 15332-2005 [37], and the results were recorded immediately after HMP heated at 160 °C for 10 min, 1 day, 2 days and 3 days.

For the chemical stability testing of HMP, a Nicolet 60SXB FTIR (Hopkinton, MA, USA) was used to obtain spectra of HMP samples after HMP heated at 160 °C with durations of 10 min, 1 day, 2 days and 3 days. The potassium bromide (KBr) disc sample preparation method was followed to take the infrared spectra. The samples were ground and mixed with KBr at a ratio of 1:99. Then, the mixer was pressed under vacuum to form pellets. FTIR spectra were recorded in a range of 4000–650 cm^−1^ at a resolution of 0.01 cm^−1^.

**Figure 2:**
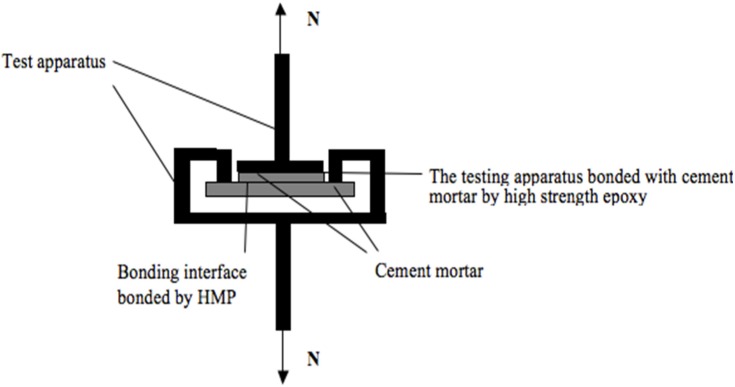
A sketch map of apparatus for bonding testing.

Mortars for the test of heating-melt adhesive strength of HMP were prepared with water to cement ratio of 0.23 and cement to sand of 0.45, and were casted in 40 mm × 40 mm × 10 mm and 70 mm × 70 mm × 20 mm mold curing for 28 days, respectively. Mortars and tensile machine were bonded by a high strength epoxy resins. The loading device for debonding was designed as shown in Figure 2. The testing HMPs were heated to the temperature at 160 °C for 1 day, 2 days and 3 days, respectively, and then immediately placed on the test mortar, and allowed to cool to ambient temperature. The adhesion performance of HMP to cement mortars after each round of heat treatment was recorded as the bonding strength readings of the machine.

### 3.3. Compatibility between HMP and Hydraulic Cement

In order to investigate the compatibility between HMP and hydraulic cement, paste were prepared with water binder ratio of 0.5 and HMP at 3% (by volume) as fine aggregate substitute. After curing for 28 days, mineralogy and chemical bonds of HMP paste after heating for 2 days are identified with aid of XRD and FTIR analyses, respectively. Properties of the paste without heating were also examined as for comparisons.

For XRD analysis, the prepared paste were broken and milled by hand in an agate mortar down to a grain size < 63 μm, and the powder was measured by XRD of Philips PW170 (Eindhoven, The Netherlands) with CuKa/40 kV/30 mA on a backloaded sample holder.

The FTIR spectra of the paste sample were recorded in the range of 4000–250 cm^−1^ using the FTIR spectrometer, as illustrated in Section 3.2.

### 3.4. Mix Design and Preparation of Mortars 

Mortar mixes were designed at a water/cement (W/C) ratio and a cement/sand (C/S) ratio of 0.23 and 1:3 by mass, respectively. HMP at 1%, 3%, and 5% (by volume) as a partial aggregate substitute were pre-blended with aggregate prior to mixing, and the corresponding mortar was denoted as “HMP-1%”, “HMP-3%” and “HMP-5%”, respectively. The plain cement mortar was denoted as control. Mixing was performed according to EN 196 testing method using a tilting drum mixer of 0.04 m^3^. Mix proportions for mortars are listed in Table 3.

**Table 3:** Mix proportions of control and HMP mortars.

Mix	Cement (g)	Sand (g)	Water (g)	W/C	HMP (g)
Control	842.1	1207.8	193.7	0.23	0
HMP-1%	842.1	1183.7	193.7	0.23	10.1
HMP-3%	842.1	1135.4	193.7	0.23	30.4
HMP-5%	842.1	1087.1	193.7	0.23	50.7

### 3.5. Fluidity, Strength and Durability Properties of HMP Blended Mortar

#### 3.5.1. Fluidity and Strength of HMP Mortars

The flow test was conducted according to the Chinese standard GB/T 2419-2005 [38]. A cone-shaped mold was placed on the center of a vibrating table and filled with freshly mixed mortar in two lifts. When the mold was removed, the vibrating table was dropped 25 times in 25 s. Then spread values on flow table were measured after the 25 drops.

For the strength tests, the mixtures were cast in 40 mm× 40 mm× 160 mm molds and demolded 24 h after casting. Then the mortar prisms were placed in a curing room temperature of 20 °C until the day of the strength tests. In order to comprehensively measure the strength and cross-check the results, both flexural and compressive strength tests were conducted at 3, 7, and 28 days. The flexure or compressive strengths measured were the average of 3 or 6 prisms of the same mixture, respectively.

#### 3.5.2. Water Permeability of HMP Mortars

The water permeability test according to the Chinese standard JGJT 70-2009 [39] was carried out on the mortar specimens after curing for 28 days. The specimens were casted from circular cylinders with top diameter of 70 mm, bottom diameter of 80 mm and height of 30 mm. With the test specimens placed inside the permeability cell, water is introduced on the top of the cell and the pressure is applied in way to force the water to penetrate through the sample. The measurement of the permeability is carried out by a method based on water penetration depth. Water with a color indicator that helps to determine the border of penetration depth is introduced.

#### 3.5.3. Initial Surface Absorption (ISA) Test of HMP Mortars

The ISA test on cylinders of size 100 mm diameter and 50 mm height was used to measure the absorptive characteristic of the surface layer of specimen in accordance with BS 1881-201:1986 [40]. After the specimens were cured for 28 days, the specimens were oven dried at 105 ± 5 °C to constant weight prior to the test. This test measures concrete permeability and its ability to absorb water during a prescribed period (ranging between 40 and 1440 min) under a head of 200 mm (7.87 inch). The rates of absorption of water at 40, 80, 120, 160, 240, 360, 720, 1440 min from the start of test were recorded. The rate of initial surface absorption is expressed in milliliters per square meter per second (mL/m^2^s).

### 3.6. Recovery of Mechanical and Durability Properties of HMP Mortars after Heat Treatment

In order to monitor the mechanical behavior of the HMP mortars after heat treatment, all mortar specimens, except the control one, were exposed to heating in an electronic furnace at temperature of 160 °C for 1, 2 and 3 days first, and then allowed to cool to ambient temperature. After each round of heating, flexural and compressive strength of mortars were determined. As for the durable properties of HMP blended mortar, the water permeability test and the ISA test were carried out on the HMP blended mortar after each heat treatment. The mechanical and durability properties of mortars after experienced various heating durations were compared with those of the mortar without heating.

### 3.7. ITZ between HMP and Cement Matrix 

The interfacial properties of HMP and cement matrix of 28-day-curing mortar (as prepared in Section 3.4) with 3% (by volume) fine aggregate replaced by HMP after heating for 2 days were characterized through a high-resolution field-effect gun digital environmental scanning electron microscope (ESEM FEG Quanta 400 from FEI Company, Hillsboro, OR, USA); using an accelerating voltage of 15 keV and a current intensity of 1 mA).

## 4. Results and Discussions

### 4.1. Heating-Melt Adhesive Strength, Melt Viscosity and Chemical Stability of HMP

The heating-melt adhesion of HMP to cement mortar is recorded as bonding strength, and the results are shown in Table 4. It is seen that the bonding strength of mortars with HMP exposed to the heating temperature at 160 °C for one two and three days becomes 100.21%, 97.98% and 96.3% of that of the mortar with HMP heating at 160 °C for 10 min, respectively. It reveals that HMP presents strong and stable adhesion property after repeated heat treatment.

The effect of heating duration on the hot melt viscosity of HMP is also shown in Table 4. It is evident that the melt viscosity of the HMP with one-, two-, and three-day heating becomes 6%, 18.3% and 23.2% higher than that of the HMP with 10-minute heating, respectively. The slight increase of melt viscosity of HMP is attributed to the ingredient of paraffin, which evaporates after prolonged exposure to heating temperature of above 160 °C.

**Table 4:** Effect of heating duration on bonding strength and melt viscosity of HMP.

Heating duration	10 min	1 day	2 day	3 day
Bonding strength (MPa)	4.7	4.7	4.6	4.5
Melt viscosity (Pa·s/180 °C)	82	87	97	101

**Figure 3:**
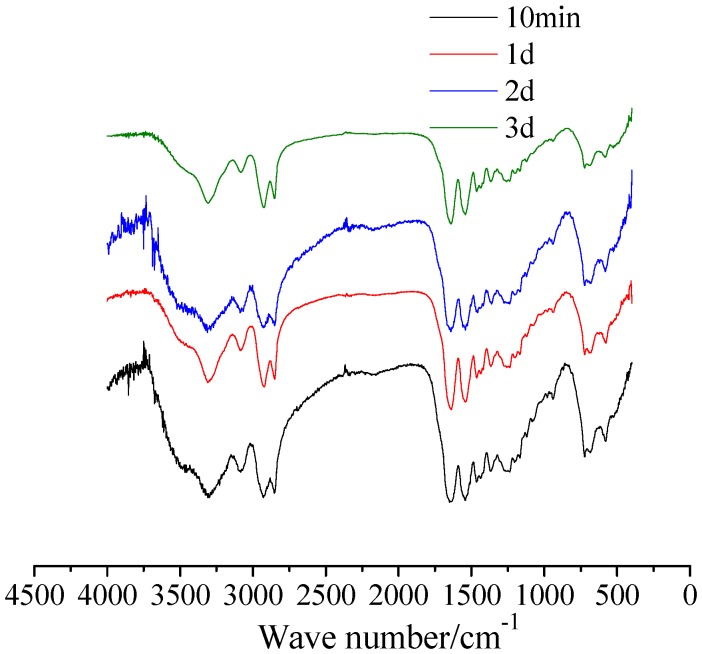
Fourier transform infrared spectra (FTIR) spectra of HMP heated at 160 °C for 10 min, one day, two days and three days.

The chemical stability of HMP after exposure to 10-minute, one-, two-, and three-day heating is monitored by FTIR. The spectra are plotted in Figure 3. It is seen that there are no new chemical bonds, which reveals no apparent oxidation reaction occurring and new substance generation in HMP after several rounds of heat treatment. Thus, we can infer that HMP presents favorable chemical stability after repeated heat treatment.

### 4.2. Compatibility between HMP and Hydraulic Cement

In order to investigate the compatibility between HMP and hydraulic cement, mineralogy and chemical bonding properties of 28-day-curing HMP blended paste are identified by means of XRD and FTIR analyses.

**Figure 4:**
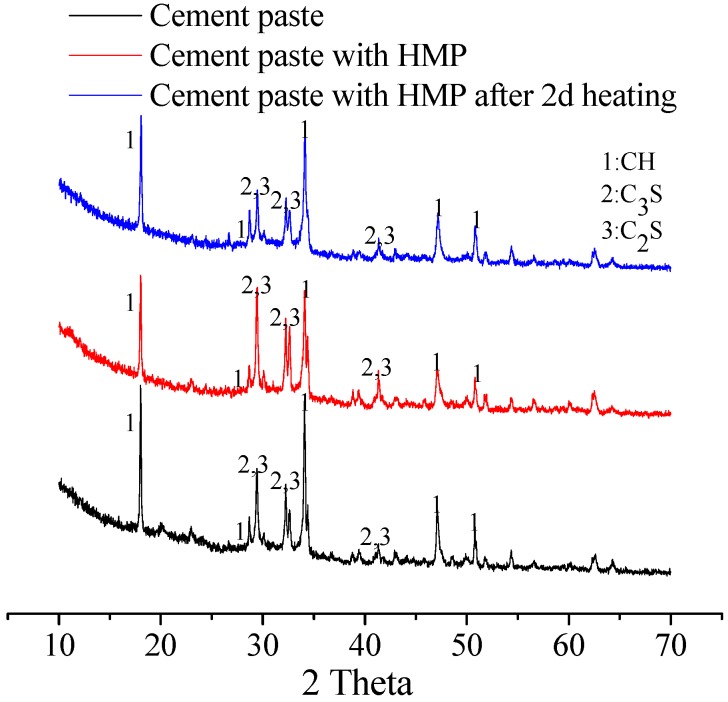
X-ray diffraction (XRD) spectra of pastes.

The XRD spectra of the cement paste, the HMP paste and the HMP paste heated for two days are plotted in Figure 4. It is seen that hardly any obvious new mineralogical phase appears in HMP pastes in comparison to cement paste. Thus, incorporation of HMP would not induce chemical reaction with cement. It is evident that cement paste with HMP heated for two days shows lower intensity at C_3_S and C_2_S (2θ = 29.3° and 32.8°) and similar height of peek at 2θ = 18.2° and 34.1° corresponding to Ca(OH)_2_ compared with that of cement paste, indicating a higher hydration degree of HMP paste after heating, which is attributed to the accelerated hydration proceeding of cement matrix after heat treatment.

**Figure 5:**
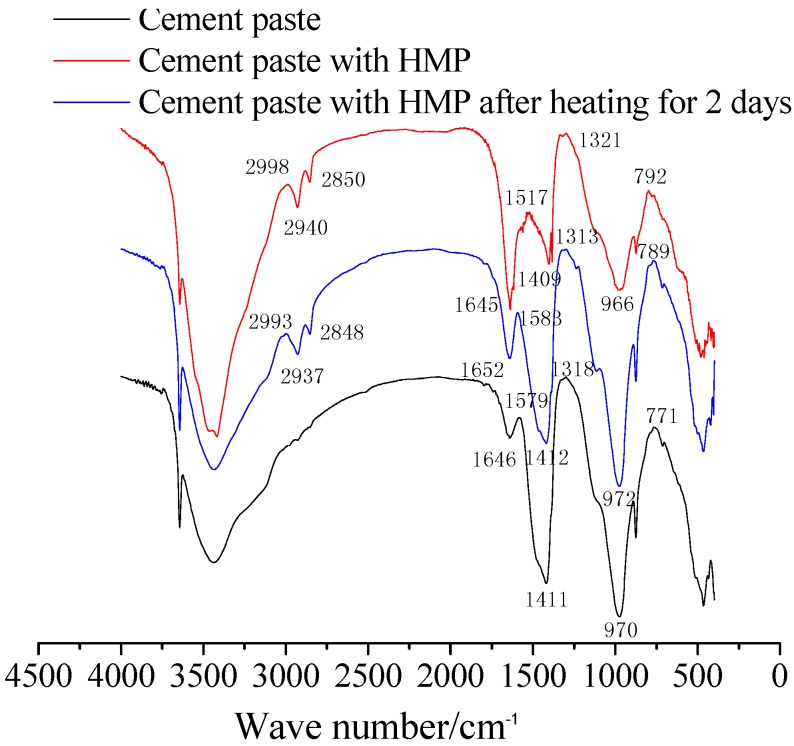
FTIR printing of pastes.

Figure 5 shows the FTIR spectra of the cement paste, the HMP paste and the HMP paste heated for two days. All paste samples exhibit strong wide bands centered around 1000 cm^−1^, attributed to the Si–O stretching vibrations of SiO_4_ tetrahedra in the C–S–H gel. All three pastes samples present a similar absorption peek value around 1000 cm^-1^ in the spectra, meaning that they possess same amorphous state. This is also confirmed by the XRD results in Figure 4.

### 4.3. Fluidity and Strength of HMP Blended Mortar

**Figure 6:**
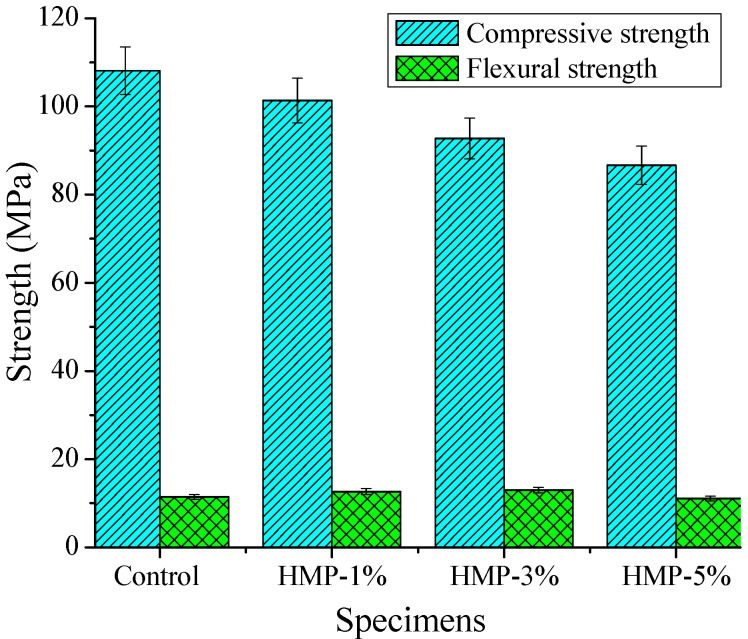
Compressive and flexural strength of control and HMP mortars.

Fluidity and strength properties of control and hardened HMP mortars are shown in Table 5 and Figure 6, respectively.

The flow diameter of fresh mortar with incorporation of 1%, 3% and 5% of sand (by volume) replaced by HMP is 3.77%, 9.43% and 13.21%, higher than that of control mortar, respectively. The increased flowability of fresh mortar with incorporation of HMP may be attributed to the two reasons: on the one hand, the surface area of HMP particles is lower than that of sand when an equal volume proportion of sand is replaced by HMP in mortars; and on the other hand, the incorporation of smooth elliptical-shaped HMP particles contributes to better fluidity compared with sand in the fresh mortars.

**Table 5:** Fluidity of control and HMP mortars.

Mixtures	Control	HMP-1%	HMP-3%	HMP-5%
Fluidity (mm)	263	277	290	300

For the compressive strength, it is seen in Figure 6 that there is a reduction in the compressive strength with increase of HMP volume. The compressive strength of mortar “HMP-1%”, “HMP-3%” and “HMP-5%” is 7.2%, 14.2% and 19.8% lower than that of the control mortar, respectively. Opposite to the compressive strength results, the flexural strength of mortar “HMP-1%” and “HMP-3%” is observed to be increased compared with the control mortar with increase in HMP volume, both are 10.6% higher than that of control mortar; whereas the flexural strength of mortar “HMP-5%” is observed to be decreased, and is 5.7% lower than that of control mortar. The phenomenon of decreased compressive strength is due to the weak bonding of interfacial transition zone (ITZ) between HMP particles and cement matrix with addition of HMP. As for the slight increase of flexural strength with increased HMP addition up to 3% of fine aggregate replacement (by volume), it may be attributed to the centroplasm theory: on one hand, HMP particles distribute in three-dimension in cement-based materials, forming a soft mass center among cement matrix and sand, which becomes the internal stress center of the composite system and leads to stress redistribution. On the other hand, HMP particles are a visco-elastic medium that can absorb and consume lots of strain energy. When the adhesion strength of HMP is lower than the bond strength of ITZ, the flexural strength began to decline.

### 4.4. Water Permeability and ISA Test of HMP Blended Mortar

The water penetration depth results of the mortar mixes against the HMP volume fraction are shown in Table 6. It is evident that with addition of HMP in mortar, the water penetration depth of mortars HMP-1%, HMP-3% and HMP-5% was increased by 16.07%, 35.71% and 49.1%, respectively, compared with that of the control mortar. That is to say, the addition of HMP would increase the water permeability of mortar. This may be attributed to weak bonding interface between HMP particles and cement matrix and hence reducing the compactness of the mortar.

**Table 6:** Water permeability results of the HMP mortars.

Mortar Specimens	Control	HMP-1%	HMP-2%	HMP-3%
Fine aggregate replacement (by volume)	0%	1%	2%	3%
Water permeability (mm)	5.6	6.5	7.6	8.4

**Figure 7:**
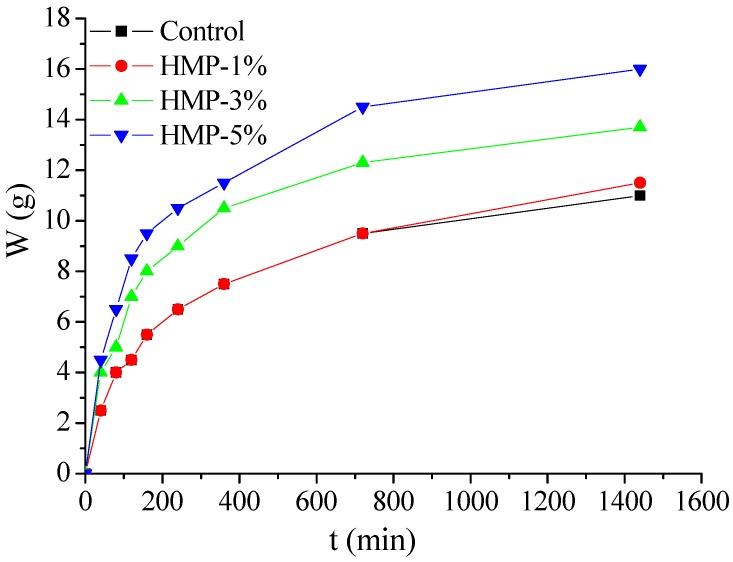
ISA test results of the HMP mortars

The results of the ISA test are shown in Figure 7. It can be seen that incorporation of HMP would increase the initial surface absorption of mortars. The water adsorption of HMP mortar increases rapidly in 6 h for all the mortars, whereas it becomes stable after 24 h. The 24-hour water penetration depth of mortars “HMP-1%, “HMP-3%” and “HMP-5%” is increased by 4.55%, 24.55% and 45.45% compared with the control mortar, respectively. It is evident that the higher addition of HMP is, the more notable increase in initial surface absorption of mortar would be.

### 4.5. Recovery of Mechanical and Durability Properties of HMP Mortars after Heat Treatment

**Figure 8:**
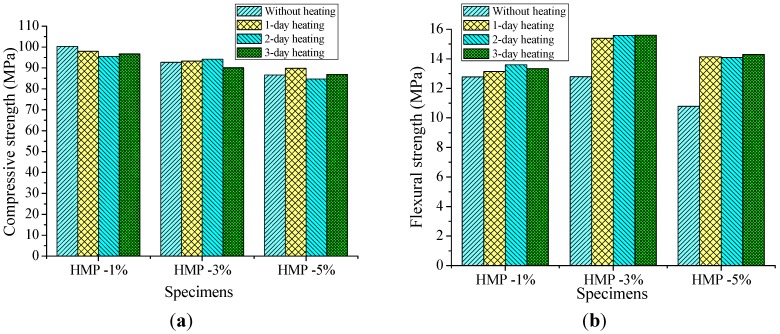
(**a**) The compressive strength of HMP mortars after heat treatment. (**b**) The flexural strength of HMP mortars after heat treatment.

After curing for 28 days, HMP blended mortars were heated at 160 °C for one, two, and three days, and then subjected to compressive and flexural strength tests. The self-repairing performance of HMP was evaluated by determining strength properties of HMP blended mortars. The compressive and the flexural strength of the heated HMP mortars are shown in Figure 8a,b, respectively.

It is seen that there is no notable change in the compressive strength for 28-day-curing HMP mortars after heat treatment compared with the ones without heating. The greatest decline in compressive strength occurs at the “HMP-1%” mortar (95.53 MPa) with two-day heating, which is 4.8% lower than the corresponding mortar (100.34 MPa) without heating. The “HMP-1%” mortar shows lower compressive strength under every heating duration compared with the corresponding mortar without heating. The compressive strength of mortar “HMP-3%” and mortar “HMP-5%” with two-day heating exhibits relatively higher values, while gradually decrease and become lower than that of the corresponding mortar without heating.

The flexural strength of all the HMP mortars experienced heat treatment is increased compared with the corresponding mortar without heating. The most notable increase rate in flexural strength appears in the group of “HMP-5%” mortars. The flexural strength of the mortar “HMP-5%” with one-, two-, and three-day heating is 31.2%, 30.7% and 32.6% higher than that of the mortar “HMP-5%” without heating, respectively. Considering the mechanical properties of HMP mortars and the repair rate of HMP, the suggestive proportion might be between 3% and 5%.

**Figure 9:**
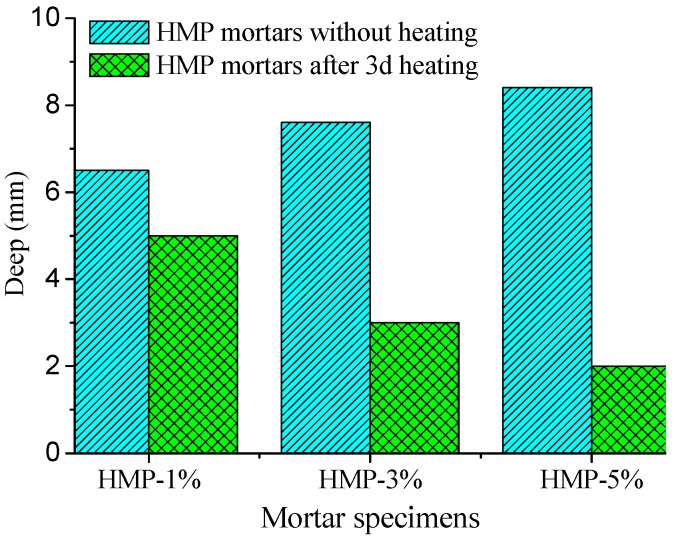
Water permeability results of the HMP mortars after three-day heating.

**Figure 10:**
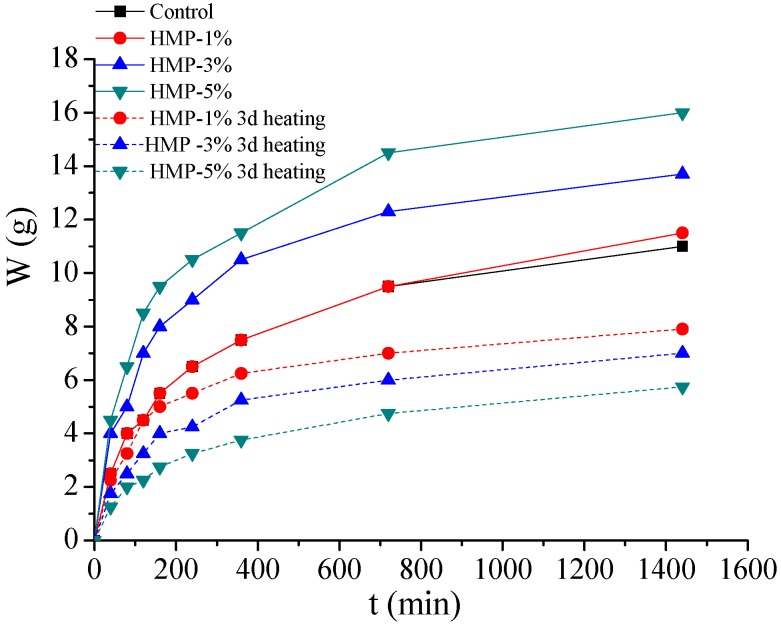
ISA test results of the HMP mortars after three-day heating.

In order to determine the recovery of durability properties of HMP blended mortar after heat treatment, the water permeability test and the ISA test were conducted on the HMP blended mortar after heating for one, two, and three days. The testing results are shown in Figure 9 and Figure 10, respectively.

It is observed that HMP mortars exhibit notable increase in water penetration resistance compared to the corresponding mortar without heating. The water permeability for “HMP-1%”, “HMP-3%” and “HMP-5%” after three-day heating decreases by 10.71%, 46.42% and 64.8% of the corresponding mortar without heating, respectively. Besides, the same trend holds for the ISA testing results. It is evident that all the HMP mortars after heating have lower than initial water adsorption after three-day heating compared with the corresponding mortars without heating. The ISA result of “HMP-1%”, “HMP-3%” and “HMP-5%” mortar is decreased by 28.18%, 36.36% and 47.73% compared with the corresponding mortar without heating, respectively.

The increased flexural strength and the increased durability of HMP mortars after heat treatment may be attributed to that the embedded HMP would be melted into liquid after the heat treatment and then fill cracks or micro-pores in the cement matrix, hence improving the compactness of the composite system.

### 4.6. ITZ between HMP and Cement Matrix 

**Figure 11:**
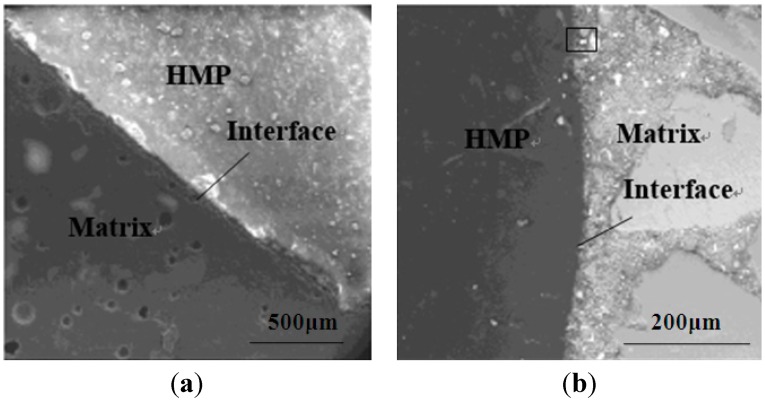
ESEM image of HMP mortars without heating and with 2-day heating. (**a**) HMP mortar without heating. (**b**) HMP mortar heated for 2 days.

The ESEM images of the ITZ of HMP mortars without heating and with two-day heating are shown in Figure 11. It is observed that a thin transparent film was observed on the surface of the HMP/cement blends without heating, whereas such a film could not be obviously recognized on image of the paste sample heated for two days, which shows only the paste matrix underneath the film. The invasion of the melt HMP into mortar matrix can be observed in the rectangular domain in Figure 10b. It reveals that addition of HMP would lead to weak bonding interface between HMP and hydraulic cement, and hence reduce the strength of the corresponding composite. However, the HMP would melt by heating for certain duration and penetrate into the voids or cracks of the hardened cement matrix as a bonding agent, which would not only well bond interfacial micro-crack in the cement matrix, but also increase compatibleness and crossing compatibility with the blend interface. Thus, the interface compatible stability and stickiness could be strengthened for the HMP/cement blends after heating and hence enhancing the compactness and strength of the HMP/cement composite system.

## 5. Conclusions

The feasibility of utilizing HMP to produce self-healing mortar was investigated. Specifically, the effect of HMP content and heating durations on properties of HMP/cement binders was assessed. Furthermore, mineralogy, phase elemental analysis and micro-structure morphology of the HMP/cement were also studied by XRD, FTIR and SEM analyses. Based on the experimental results, the following major conclusions can be drawn:

(1) Incorporation of HMP contributes to improve the workability of mortar, however it leads to a decrease of compressive strength. The compressive strength of mortars decreases with increase in volume percentage of aggregate replacement by HMP. The compressive strength of mortars “HMP-1%”, “HMP-3%” and “HMP-5%” is 7.2%, 14.2% and 19.8% lower than that of control mortar, respectively. Opposite to the compressive strength results, the flexural strength of mortar “HMP-1%” and “HMP-3%” is observed to be increased compared to control mortar, both are 10.6% higher than that of control mortar; whereas the flexural strength of mortar “HMP-5%” is observed to be 5.7% lower than that of control mortar. The permeability of mortar increases with the increase of HMP content due to the weak bonding of ITZ between HMP particles and cement matrix.

(2) The XRD and FTIR analyses of the control paste and the HMP pastes with heat treatment confirm that no obvious chemical reaction occurs between HMP and hydraulic cement, and incorporation of HMP would not inhibit cement hydration proceeding in the cement matrix after heat treatment.

(3) There is no obvious change in the compressive strength for HMP mortars after three rounds of heat treatment, whereas the flexural strength of all the HMP mortars that experienced heat treatment present notable increases compared with the corresponding mortar without heating. The most remarkable increase in flexural strength appears in the group of “HMP-5%” mortars. The flexural strength of the mortar “HMP-5%” with one-, two- and three-day heating is 31.2%, 30.7% and 32.6% higher than that of mortar “HMP-5%” without heating, respectively. The water permeability of mortars “HMP-1%”, “HMP-3%” and “HMP-5%” after three-day heating decreases by 10.71%, 46.42% and 64.8% of the corresponding mortar without heating, respectively. The ISA result of “HMP-1%”, “HMP-3%” and “HMP-5%” mortar is decreased by 28.18%, 36.36% and 47.73% compared with the corresponding mortar without heating, respectively. Addition of HMP exhibit notable decreases in water penetration and ISA compared with the corresponding mortar without heating.

(4) The increased addition of HMP could lead to continuously decreased compressive strength and durability of mortar specimens. However, the flexural strength of specimens could be slightly improved with incorporation of HMP up to 3% (by volume) as substitute for fine aggregate. With addition of HMP, there are significant improvements in flexural strength and durability of HMP mortars after heat treatment compared with those of control mortar. Thus, HMP presents excellent crack healing properties. Considering the economic factors, mechanical improvements of HMP mortars and the repair rate of HMP, the suggestive fine aggregate replacement (by volume) by HMP might be between 3% and 5%. The ESEM observation on ITZ of HMP mortar further demonstrates that the bonding between HMP and hydraulic cement may be mainly due to physical adhesion rather than chemical bonding.
